# Characterization of Reduced-Fat Mayonnaise and Comparison of Sensory Perception, Rheological, Tribological, and Textural Analyses

**DOI:** 10.3390/foods11060806

**Published:** 2022-03-11

**Authors:** Christopher N. Schädle, Stephanie Bader-Mittermaier, Solange Sanahuja

**Affiliations:** 1 Aroma and Smell Research, Department of Chemistry and Pharmacy, Friedrich-Alexander University Erlangen-Nürnberg, Henkestraße 9, 91054 Erlangen, Germany; 2 Department of Food Process Development, Fraunhofer Institute for Process Engineering and Packaging IVV, Giggenhauser Str. 35, 85354 Freising, Germany; stephanie.mittermaier@ivv.fraunhofer.de; 3 School of Agricultural, Forest and Food Sciences (HAFL), Bern University of Applied Sciences, Länggasse 85, 3052 Zollikofen, Switzerland; solange.sanahuja@bfh.ch

**Keywords:** tribology, rheology, emulsion, lubricity, viscosity, spreadability, calorie-reduced, dietary fiber, corn dextrin

## Abstract

Reduced-fat products can help to fight obesity and its associated health risks. To develop appealing products, both product-specific fat replacers and suitable analytical methods for the characterization of fat-associated properties are important. The rheology, tribology, texture, and spreadability of a reduced-fat mayonnaise with different concentrations of corn dextrin were analyzed to determine properties such as flow behavior, viscosity, lubricity, firmness, and stickiness. Additionally, a sensory panel analyzed the samples for their mouthfeel (creaminess, firmness, and stickiness). Correlations between the results of the instrumental methods suggested that the analytical effort for the future development of appealing reduced-fat food products can be reduced. In addition, several correlations were identified between the instrumental and the sensory data. Results from tribological measurements correlated with the sensory attribute of stickiness, suggesting that tribometry can complement or constitute an alternative to complex and expensive human sensory tests. Additionally, the use of Stevens’ power law showed a high correlation between the Kokini oral shear stress and the sensory attribute of creaminess. The instrumental texture properties (firmness, stickiness) also correlated with the sensory sensation. The identified correlations obtained from comparing different methods may help to estimate the possible applications of new fat replacers and facilitate innovative product development.

## 1. Introduction

Obesity is one of the dominant worldwide diet-related health concerns and can cause high blood pressure, type 2 diabetes mellitus, coronary heart disease, stroke, certain cancers, and degenerative diseases [1]. Food reduced in sugar, fat, or calories, along with sufficient exercise, can contribute to combating obesity and reducing the associated health risks. However, a large number of reduced-fat products face rejection because the consumers connect these products with poor organoleptic qualities [2,3]. New approaches are therefore required to provide high-quality (or innovative combinations of) fat replacers, ideally based on vegetable proteins, dietary fibers, and hydrocolloids.

Fat affects a variety of characteristics in food, such as nutritional, technological, and sensory properties, including appearance, flavor, aroma, color, lubricity, texture, and mouthfeel [4,5]. The development of reduced-fat products is often application-related and based on trial and error, due to insufficient methods for the objective evaluation of the fat-typical properties of fat replacers. One analytical method usually covers only one specific aspect of fat properties, and real conditions during consumption, such as the oral process, cannot be mimicked. Systematically recorded data and evaluation methods for assessing the application potential of new fat substitutes are scarce, which considerably hinders the development of new vegetable fat replacers. In addition to developing new methods, the correlations between the analytical results from various measurements need to be investigated to build up a toolbox of appropriate methods that can sufficiently characterize a given fat replacer in a food model system and serve to evaluate and predict its application potential.

Corn dextrin (CD) is a fat replacer made from corn starch that is partially hydrolyzed. It is categorized as a water-soluble dietary fiber or resistant dextrin. Its average molecular weight is 5 kDa, and it consists of 82 to 88% of dietary fibers, 0.3% mono- and disaccharides and <0.1% starch, and has a dextrose equivalent value of DE 3–5 [6]. Additionally, it presents many benefits, such as a low calorific value of 8.4 kJ/g (2 kcal/g) compared to fat with 37.6 kJ/g (9 kcal/g), a neutral taste, a small effect on viscosity, and can be labeled as dextrin [7], meeting current consumer demands for clean labels [8]. Furthermore, it has been identified in previous internal trials as a promising fat replacer in emulsions and dairy products [9]. The maltodextrin (Malt) used in this study exhibits a dextrose equivalent of DE 20. Like CD, it is made of starch, but it differs from CD with regard to the glycosidic bond distribution (Table 1) [10], which is why CD is classified as a dietary fiber and Malt is not.

Rheological and textural properties are known to affect the mouthfeel of food emulsions, such as mayonnaise. As the gap between tongue and palate is rather large in the front of the oral cavity [11], the oral perception is initially dominated by bulk properties. Upon chewing, mixing with saliva, and squeezing between the moving surfaces of the tongue and palate, the perception depends more on thin film rheological behavior, which can be analyzed by tribological measurements. Additionally, sensory attributes such as creaminess correlate with the lubrication properties of mayonnaise and other emulsions, which were also determined using tribology [12,13]. Tribology is the study of the friction of surfaces in relative motion [14], and its prevalence in food science has increased in recent years. However, the best methodology for food tribometry is still not clear from the studies in the literature. The results of tribological measurements are typically presented in the Stribeck curve that displays the coefficient of friction (COF) as a function of sliding speed. According to Pondicherry et al. [15], the extended Stribeck curve is divided into the static and kinetic regions. The latter is subdivided into the boundary friction, the mixed friction, and the (elasto-) hydrodynamic friction regime. Researchers estimated a speed of up to 200 mm∙s^−1^ for the movement of the tongue during oral processing [16]. To evaluate the tribological measurements, Agyei-Amponsah et al. [17] selected the friction values at 5 and 100 mm∙s^−1^. At these speed levels, the lubricant fluids were effectively entrained in the mixed and hydrodynamic regime. While the authors determined no significant correlation between sensory and tribology results in the mixed regime at 5 mm∙s^−1^, they linked lower friction values at 100 mm∙s^−1^ with higher values in the sensory ratings of oiliness and creaminess in reduced-fat mayonnaise-type emulsions. Similar correlations were found by Dresselhuis et al. [18]. They suggested that their tribology and sensory results were based on the different coalescence behavior of the emulsions. Several researchers found that an increase in fat content increases the overall lubricity of the tribological system, resulting in a decreased friction [19,20,21,22]. Authors also described the importance of oral friction processes in determining the sensory properties of food [23,24].

Mayonnaise is a popular, but high caloric, sauce that is high in fat [25]. Many researchers examined the effect of fat replacers in reduced-fat mayonnaise and salad dressings [17,26,27,28,29], but the correlation of instrumental and sensory measurements is still inadequate due to the complex food matrix and differences in human sensory perception. Various research groups worked with starch- and flour-based products as fat replacers in mayonnaise [28,29,30,31] and identified them as potential and viable fat replacers. However, these fat replacers exhibited a few shortcomings, e.g., they changed product characteristics like color, viscosity, and the elastic and loss moduli, and fat could only be reduced to a certain level. CD might help overcome these deficiencies.

In this study, we reformulated a model salad mayonnaise with 50% fat, substituting fat with either water, different concentrations of CD, or Malt. Possible correlations between the results of different measurement methods were investigated with the aim of reducing the effort for developing new reduced-fat foods. Most importantly, tribological measurements, which are currently only used to a minor extent in food characterization, were introduced, as they can complement or constitute a viable alternative to extensive application and sensory tests. This may help to estimate the possible applications of new fat replacers and facilitate innovative product development.

## 2. Materials and Methods

### 2.1. Materials

Vegetable fat (Rapso rapeseed oil, VOG AG, Linz, Austria), sugar (fine white refined sugar, Suedzucker AG, Mannheim, Germany), table salt (Salta Siede-Speisesalz, Suedwestdeutsche Salzwerke AG, Heilbronn, Germany), mustard (Thomy Delikatess Senf Mittelscharf, Nestle Deutschland AG, Frankfurt am Main, Germany), vinegar (Tip Tafelessig, real GmbH, Dusseldorf, Germany), skim milk powder (Backstars.de–Inh., Andreas Zoellner, Bellenberg, Germany), citric acid and xanthan gum (both Wuerzteufel GmbH, Nagold-Hochdorf, Germany) were obtained from retailers. Corn dextrin (CD) (NUTRIOSE FM 06, Roquette Frères, Lestrem, France), sunflower protein (Heliaflor 55, AOT GmbH, Wiggensbach, Germany), corn starch (NOTAVATION LUMINA 300, Ingredion GmbH, Hamburg, Germany), and maltodextrin (Malt) (MD20, Avebe GmbH, Karstädt, Germany) were kindly donated by the suppliers.

### 2.2. Manufacturing of the Mayonnaise

The mayonnaise was prepared using a laboratory reactor system (IKA-Werke GmbH & Co. KG, Staufen, Germany) equipped with a dispenser (Ministar 40 digital, IKA-Werke GmbH & Co. KG, Staufen, Germany) and an Ultra-Turrax (T 25 digital, IKA-Werke GmbH & Co. KG, Staufen, Germany). An external water bath was preheated to 95 °C. The formulations are summarized in Table 2. Initially, water (45 °C) was poured into the reactor. Using the dispenser (120 rpm) and the Ultra-Turrax (10,500 rpm), the premixed dry ingredients (sunflower protein, corn starch, sugar, salt, citric acid, and depending on the formulation: skim milk powder, CD, Malt, and xanthan gum) were slowly added. Two minutes after the addition of the dry ingredients and continuous stirring and dispersing, the oil was added very slowly. After oil addition, the mixture of vinegar and mustard was added, and the speed of the dispenser was increased to 150 rpm. After another two minutes of stirring and dispersing, the preheated water bath was connected to the laboratory reactor and the mayonnaise was heated. Once it reached a temperature of 72 °C, the water bath was disconnected again, and the mayonnaise was stirred and dispersed for another 10 min. The hot mayonnaise was poured into glass jars, sealed and left on the lid at room temperature for 5 min. Subsequently, the mayonnaise samples were stored at 1 °C for at least one week and then kept overnight at 5 °C before being analyzed. On the day of analysis, the mayonnaise was gently stirred with a spatula and then stored at 5 °C for at least another 30 min before the first measurements were performed. For each formulation, three batches of mayonnaise were prepared (875 g mayonnaise per batch).

It was necessary to achieve different gradations of the properties, rather than matching the properties of the full-fat salad mayonnaise. However, the gradations had to be small enough for panelists not to perceive differences at each concentration level, to avoid making the differences too obvious. To stabilize the reduced-fat samples, the recipe was adapted accordingly, e.g., by adding xanthan gum and skimmed milk powder. The resulting changes in the formulations in comparison to the full-fat samples were considered.

### 2.3. Composition and pH

The dry matter content of the mayonnaise samples was determined according to AOAC standards [32], with a thermo-gravimetrical system at 105 °C (TGA 701, Leco Instrumente GmbH, Mönchengladbach, Germany). The protein content was calculated based on the nitrogen content determined according to the Dumas combustion method as described by AOAC standards [33] using a TruMac N Nitrogen Analyzer (Leco Instrumente GmbH, Mönchengladbach, Germany) and a conversion factor of N × 6.25. The fat content was determined based on the Caviezel method, DGF C-III 19 (00) [34], with slight modifications. In addition to the Caviezel method, the fats were derivatized with trimethylsulfonium hydroxide prior to being analyzed using gas chromatography. The pH of the mayonnaise samples was measured using a 206-pH2 digital pH meter (Testo SE and Co. KGaA, Lenzkirch, Germany).

### 2.4. Texture and Spreadability

The firmness and stickiness (texture) and the spreadability were determined using a TA.XTplusC Texture Analyzer (Stable Micro Systems, Godalming, UK) with a 10 kg load cell at room temperature. To analyze the texture, the mayonnaise (5 °C) was filled into a tube (inner diameter of 26 mm, height 35 mm) and smoothed off at the top. A plastic cy-linder with a diameter of 12.7 mm (P/0.5–½” Dia Cylinder Delrin, Stable Micro Systems) was used as measurement probe that entered the sample at a speed of 1 mm∙s^−1^ for a distance of 10 mm and then returned to the starting position with the same speed. The firmness is defined as the positive peak force of the penetration and the stickiness as the negative peak force at the withdrawal of the probe (resistance force). Example curves of the measurements can be found in the supplementary materials (Figure S1). Each sample was measured at least seven times. To analyze spreadability, the texture analyzer was equipped with the TTC Spreadability Rig (Stable Micro Systems). The product cups were filled with mayonnaise (5 °C), smoothed on top, and analyzed as described by Javanmard et al. [35]. The male cone entered at a speed of 3 mm∙s^−1^ and then moved back to the starting position at 10 mm∙s^−1^. The work of shear is determined as the area under the curve of the positive peak. Smaller values mean easier spreadability, since a more easily spreadable products require a smaller force to be driven out of the cup by the male cone [36]. The area of the negative peak during the withdrawal of the upper cone indicates the work of adhesion. Example curves of the spreadability measurements can be found in the supplementary materials (Figure S2). Each sample was measured at least seven times.

### 2.5. Rheological Properties

The rheological behavior of the mayonnaise samples was measured using a Physica MCR 301 controlled shear strain rotational rheometer (Anton Paar Germany GmbH, Ostfildern, Germany), equipped with a concentric cylinder geometry (CC27, d = 27 mm) and with a Peltier temperature control (C-PTD200). The system was tempered to 5 °C after 20 g of the mayonnaise had been filled into the cup. A rest time of 5 min allowed additional loading stresses to dissipate. Flow curves were determined at 5 °C in three steps, 4 min each, by linearly increasing the shear rate (0.5–200 s^−1^), holding (200 s^−1^), and linearly decreasing the shear rate (200–0.5 s^−1^) and observing the resulting shear stress. Example curves of the rheological measurements can be found in the supplementary materials (Figure S3).

The rheological properties of the mayonnaise samples were characterized using the Herschel–Bulkley model (Equation (1)) for non-Newtonian fluids by increasing the shear rate from 0.5–200 s^−1^, because it is commonly used to describe the flow properties of emulsion systems like mayonnaise [37]:

(1) $$\tau =\tau_{0}+K\times {\dot{\gamma }}^{n}\;$$

where *τ* is the shear stress (Pa), $\tau_{0}$ is the yield stress (Pa), $\dot{\gamma }$ is the shear rate (s^−1^), *K* is the consistency index (Pa∙s*^n^*), and *n* is the flow index (–). The Herschel–Bulkley parameters were used to calculate the Kokini oral shear stress *τ* (Kokini OSS) [38] (Equation (2)):

(2) $$\tau =\tau_{0}+Kv^{n}{\left(\frac{1}{{h_{0}}^{\frac{\left(n+1\right)}{n}}}+{\left(\frac{F}{R^{n+3}}\times \frac{n+3}{2\pi K}\right)}^{\frac{1}{n}}\times \frac{\left(n+1\right)t}{2n+1}\right)}^{\frac{n^{2}}{\left(n+1\right)}}\;$$

with the velocity of tongue *v* = 2 cm∙s^−1^, normal force *F* = 1 N, radius of plug *R* = 2.5 cm, time *t* = 1 s, initial plug height *h*_0_ = 0.2 cm, and the Herschel–Bulkley parameters yield stress $\tau_{0}$, consistency index *K*, and flow index *n*. Cook et al. [39] used Kokini OSS as a measure of in-mouth viscosity. Furthermore, the apparent shear viscosity at 10 s^−1^ and 100 s^−1^ were determined and the hysteresis loop was obtained using the flow curves of steps 1 and 3, i.e., by the increasing and decreasing shear rate. The hysteresis area was calculated by $A=\left(A_{up}-A_{down}\right)$, where $A_{up}$ is the area under the flow curve of step 1 and $A_{down}$ of step 3 [40], and this was used to determine the time-dependent rheological effects of the mayonnaise samples. Three measurements were carried out for each sample and no wall slippage or synaeresis was observed during the measurements.

### 2.6. Tribological Properties

The tribological properties of the mayonnaise samples were measured with the rheometer described above, equipped with a tribology measuring cell with Peltier temperature control (T-PTD200, Anton Paar Germany GmbH, Ostfildern, Germany), Peltier hood (H-PTD200), measuring shaft (BC12.7), and sample holder (SH-BC6). To simulate the interaction of the tongue and palate in the mouth, a ball-on-three-pins set-up was used, with a glass ball (soda lime glass, 12.7 mm in diameter) and three polymeric pins (polydimethylsiloxane (PDMS), cylinder, 6 mm in height and diameter), the so-called tribo-pair, with a deflection angle of the pins of 45°. An aliquot of 1 g mayonnaise was used and the temperature in the cell was constantly 37 °C, representing the average temperature in the human mouth. The glass ball was lowered onto the pins with a normal force of 1 N and subsequently, the mayonnaise was distributed by turning the ball for 2 s at a speed of 0.05 min^−1^. The in-mouth force was reported to be between 0.01 and 10 N [41]; Flamminii et al. [12] used a normal force of 1 N and Nguyen et al. [42] used 1 N and 2 N in tribological measurements as a moderate normal force applied on emulsion food during oral processing. Since the normal force in our setup was divided over three pins and with an angle of 45°, the force perpendicular to the surface of each pin was about 0.24 N. After a 5 min rest period, allowing additional loading stresses to dissipate, the sliding speed was increased logarithmically from 10^−6^ mm∙s^−1^ to 10^3^ mm∙s^−1^, and the corresponding coefficient of friction (COF) was determined. Per measurement, the sequence of resting and increasing the sliding speed was performed twice more, without replacing the sample, resulting in three curves of the COF versus the sliding speed (referred to from hereon as curve 1, 2, and 3). Between the measurements of the samples of the same sample batch, the residues in the sample holder were removed carefully with a tissue and water. The sample holder and the pins were dried with compressed air, rinsed with ethanol, and again dried with compressed air before a new sample was loaded. Each sample batch was measured four times and with a new set of three PDMS pins for each batch.

### 2.7. Selected Sensory Attributes

The mayonnaise samples were analyzed according to DIN ISO 8587:2006 [43]. The panelists (*n* = 12, 8 females and 4 males) were recruited from an established sensory panel, which has received long-term training in sensory testing. All panelists felt able to perform the sensory evaluation; none of them reported any known illnesses. The nine different samples were labeled with random three-digit numbers and offered to the assessors in small glass beakers. The evaluation was performed at room temperature and under normal light conditions. It was carried out individually with no interaction between panelists. The panel leader demonstrated how much mayonnaise to put on the spoon to ensure all panelists assessed the same amount. Panelists were asked to rank the samples according to the attributes of firmness, stickiness, and creaminess in individual sessions with a 5 min break between each session. Water was provided for mouth rinsing in between. Panelists were offered definitions of each attribute taken from current literature on the sensory profiling of fat-replacer samples [44]. Firmness is defined as the force required to squeeze the mayonnaise specimens between the palate and the tongue. Stickiness, in turn, describes the force required to remove the sample from a surface, in this case the palate, and creaminess is the degree of creamy texture perceived in the mouth.

### 2.8. Data and Statistical Analysis

The Shapiro–Wilk test was used to confirm the Gaussian distribution, and Levene’s test was applied to test for the homogeneity of variance. The data were subsequently processed by a single factor (univariate) analysis of variance (ANOVA) followed by Tukey’s honest significance test (*α* = 0.05). The data are expressed as mean ± standard deviation.

The results of the sensory analysis were evaluated by the Friedman analysis (*α* = 0.05) and by computing the rank sum for each sample. The Friedman’s statistic was calculated with Equation (3) [43]:

(3) $$\chi_{r}^{2}=\frac{12}{n\times k\times \left(k+1\right)}\overset{k}{\underset{i=1}{\sum }}R_{i}^{2}-3\times n\times \left(k+1\right)$$

where *n* is the number of panelists, *k* is the number of samples, and *R* is the rank sum for each sample. Experimental $\chi_{r}^{2}$ values were compared with the critical values of $\chi_{crit}^{2}$ with (*k* − 1) degree of freedom, which is $\chi_{crit}^{2}$ = 15.15 for *α* = 0.05, *n* = 12, and *k* = 9 [45]. Significant differences between samples were determined with the LSD (Least Significant Difference) according to the DIN ISO 8587:2006 [43]. Differences between rank sum scores of two mayonnaise samples were considered as significant if they exceed LSD = 25.2 (for *α* = 0.05, *z* = 1.96, *n* = 12, and *k* = 9) [43].

For the correlation of the results, the Pearson correlation coefficient between the parameters was determined. The correlation with the sensory data was also evaluated taking the Stevens’ power law in account, since sensory perception does not always develop linearly such as physical quantities often do; thus, the correlation between instrumental and sensory measurements is not always linear. The instrumental and the sensory data sets were fitted to Stevens’ psychophysical power law [46] (Equation (4)):

(4) $$\Psi =k\phi^{\beta }\;$$

with the sensory magnitude Ψ, the instrumental index value ϕ, the constant *k* that depends on the measurement units, and the exponent *β* representing the curvature of the power function. *β* = 1 indicates that the perceived sensory sensation varies in a linear form with the stimulus intensity of the instrumental measurement; *β* > 1 indicates that the sensation magnitude increases more rapidly than the stimuli intensity; and *β* < 1 indicates that the sensation magnitude increases less rapidly than the stimuli [47]. The correlation coefficient *r* was determined using the sensory magnitude of the sensory test and the calculated sensory magnitude of the fitted Stevens’ power law function.

## 3. Results and Discussion

### 3.1. Composition and pH

The dry matter content for the full-fat sample was 59.5 ± 0.8 g/100 g and increased from 41.3 ± 0.2 g/100 g at 0% CD to 50.7 ± 0.3 g/100 g at 8% CD, because the CD concentration was balanced with water in the reduced-fat formulations. The fat content was 50.9 ± 1.7 g/100 g in the full-fat sample and was reduced by around 50% in the reduced-fat samples. The protein content was 1.1 ± 0.1 g/100 g in the full-fat sample and 2.6 g/100 g in all reduced-fat samples. When calculating the energy densities of the samples, the values differed depending on the composition of each formulation. The full-fat sample exhibited the highest energy density of around 1816 kJ/100 g (441 kcal/100 g). Comparable commercially available full-fat salad mayonnaise (50–54% fat) demonstrate energy densities in the range of 1870 kJ/100 g (454 kcal/100 g) to 2270 kJ/100 g (551 kcal/100 g). The sample with 0% CD exhibited the lowest energy density, with about 1057 kJ/100 g (256 kcal/100 g). With increasing CD concentrations, the energy density increased from 1061 kJ/100 g at 0.5% CD to 1124 kJ/100 g at 8% CD, thus a reduction range in the energy density of around 38% to 42% was reached when compared to the full-fat sample. The sample with 8% Malt demonstrated an energy density of 1184 kJ/100 g (286 kcal/100 g). The effects of diverging compositions on the rheological, tribological, and textural characteristics are discussed in more detail in the upcoming sections. A table with all values for fat, protein, and dry matter content, as well as the energy densities, can be found in the supplementary materials (Table S1). The pH values of the reduced-fat mayonnaise samples were around pH 4.6. Only the full-fat sample demonstrated a significant difference, with a value of pH 4.2.

### 3.2. Texture and Spreadability

Firmness and stickiness (Table 3) are important textural attributes influencing the mouthfeel [48] and processability of the product. The highest firmness was found for the mayonnaise samples with more than 4 g/100 g CD and for the Malt sample. The higher the absolute value of the stickiness (the more negative the value), the stickier and more cohesive the mayonnaise [37]. Both firmness and stickiness initially increased with the amount of CD added, but did not change significantly for concentrations ≥4 g/100 g. At the same time, the water content in the samples decreased with increasing CD concentrations, which could explain the initial increase in firmness and stickiness also accompanying increasing dry matter content. Carcelli et al. [29] also found increasing firmness and stickiness values with increasing dry matter content in reduced-fat mayonnaise samples, which is in agreement with our results at lower CD concentrations. Since the 8% CD and 8% Malt samples match for both characteristics, the differences in their chemical structure did not appear to differently affect the firmness and stickiness of the samples.

The work of shear describes the physical work required to spread the mayonnaise, e.g., on a sandwich or hamburger bun, and may be related to in-mouth spreadability (Table 3). The values increased with increasing CD concentrations, i.e., the mayonnaise got more resistant to a knife during spreading. The work of adhesion of the mayonnaise samples, i.e., the adhesion of the sample to the knife used for spreading, also increased to a similar extent, which is indicated by decreasing values for the work of adhesion (N∙s^−1^). Those results revealed that with higher CD concentrations, firmer and stickier samples were obtained. The sample where 8 g/100 g Malt was added in the formulation behaved similar to the 8% CD sample. Despite our results that texture and spreadability develop in the same manner depending on the formulation, other authors noted that the firmness and spreadability of spreads are not always related in this way, and a higher firmness of the sample does not always mean a higher work of shear [36]. Therefore, we used both methods to characterize our mayonnaise samples.

### 3.3. Rheological Properties

Yield stress ($\tau_{0}$), flow index *n*, and consistency *K* of the Herschel–Bulkley model (Equation (1)) are shown in Table S2. The yield stresses of the reduced-fat samples were significantly higher than those of the full-fat sample. The yield stress is a suitable factor to assess mayonnaise, especially when used in salad dressing, because it determines its ability to adhere to the salad surface [30], which was improved in our reduced-fat samples. Additionally, the level of yield stress determines the stability in low stress situations, such as storage and transportation, the spreadability at the time of utilization, the perceived thickness or creaminess during mastication, as well as the product behavior during mixing, stirring, and pumping during the manufacturing process [49,50,51], which were also improved in our reduced-fat samples.

All mayonnaise formulations were non-Newtonian liquids with shear-thinning flow (*n* < 1), and no noteworthy differences were found between our samples. Agyei-Amponsah et al. [17] also found shear-thinning properties for full-fat and reduced-fat mayonnaises in their study. However, like other authors [30,52], they also found a decrease in the flow index with an increasing fat replacement level. This is due to the kind of fat replacers they used, which were different modified starches. Starches have a weight-average molecular weight of around 51 MDa for corn starch, or even over 83 MDa for rice starch [53]. In contrast, the CD used in this study has a molecular weight of 5 kDa [54]. Therefore, the differences between our investigations and the previous studies can be attributed to the use of low molecular weight CD. Besides, shear-thinning can be caused by a breakdown of the structure during a shearing process [55]. Moreover, the particles and long structural units orientate along the shear direction, minimizing the resistance of flow. Because CD in the mayonnaise samples is a small molecule in comparison, the shear impact and the alignment of the molecules in the shear field is negligibly low; therefore, the CD concentration had no effect on the flow index.

**Figure 1:**
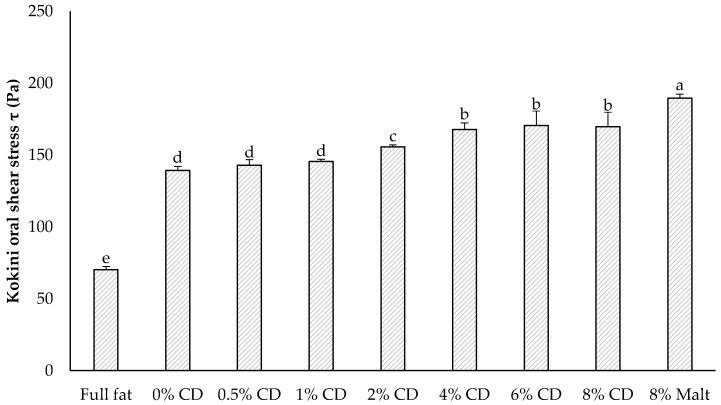
The Kokini OSS of all mayonnaise formulations. The data are expressed as mean ± standard deviation (*n* = 9). Bars with different letters indicate significant differences between samples (*p* < 0.05) following one-way ANOVA (Tukey).

Two possible instrumental indicators of perceived thickness of food formulations were calculated based on previous works. Viscosity was calculated at a shear rate of 10 s^−1^ and 100 s^−1^ (Table S2), since these values are assumed for chewing and swallowing processes [56] and can be associated with the viscosity of semisolid products perceived in the mouth [47,57,58]. Tárrega et al. [40] found a relationship between the viscosity at 10 s^−1^ and the mouthfeel for a vanilla desert, and Krzeminski et al. [57] used a shear rate of 100 s^−1^, as this was the best way to assess the oral perception of semisolid dairy products. In addition, Kokini et al. [59] proposed a physical index of in-mouth thickness based on the change in shear stress during eating. Due to the similar informative value, only Kokini OSS is shown in Figure 1, whereas the viscosities (Table S2) can be looked up in the supplementary materials. The Kokini OSS was originally based on the Ostwald–de Waele model and was later extended by Stokes [38] to include the yield stress, in order to adapt the Kokini OSS to the Herschel–Bulkley model (Equation (2)). The Kokini OSS was the lowest for the full-fat sample and increased with the increase of the CD concentrations in the reduced-fat samples (Figure 1).

The mayonnaise samples can be roughly divided into clusters, with the full-fat sample at the lowest level according to its low Kokini OSS value compared to the other samples. The reduced-fat mayonnaises with 0, 0.5, and 1 g/100 g CD exhibited similar Kokini OSS values with about 150 Pa. At a concentration of equal to or above 2 g/100 g CD, the Kokini OSS increased again, revealing a plateau with a maximum of 170 Pa at 4, 6, and 8 g/100 g CD, respectively. This increase in Kokini OSS can most probably be attributed to increasing dry matter contents, since CD replaced water in the reduced-fat formulations. Similar to our investigations, Lefranc-Millot et al. [7] also found that CD only has a minor impact on viscosity [7], supporting the fact that the increase was caused by the changes in the dry matter content. The sample with 8 g/100 g Malt exhibited the highest Kokini OSS of all mayonnaises, which was unexpected, since the viscosity of dextrins usually decreases with the increase of their dextrose equivalent value [60,61,62]. Additionally, CD is a much more branched molecule than Malt, which is known to foster an increase in viscosity. The large deviation of the full-fat sample was expected because of the differences in the formulations, such as the use of skim milk powder and xanthan gum as stabilizers in the reduced-fat samples, as well as the slightly higher amount of corn starch in these samples. An emulsion will become highly unstable if the fat content is reduced below a certain critical level, but can be stabilized by more intensive homogenization conditions to reduce the droplet size, or by the addition of stabilizers and thickening agents, such as starch and gums [63], as was the case in our reduced-fat samples. Raymundo et al. [64] described a decrease in the Sauter mean diameter in low-fat mayonnaise with increasing xanthan gum concentrations, and according to Katsaros et al. [65], the yield stress, consistency, and apparent viscosity of mayonnaise increase with a decreasing size of oil droplets. Therefore, in the reduced-fat-samples, the increase in the Kokini OSS compared to the full-fat sample was not only due to the xanthan gum concentration, but also to smaller oil droplets caused by the xanthan gum. The same phenomenon likely occurred with other ingredients such as corn starch, as Agyei-Amponsah et al. [17] found that replacing sunflower oil with modified corn starch produced a reduced-fat mayonnaise with higher viscosity and smaller oil droplets compared to full-fat mayonnaise.

### 3.4. Tribological Properties

**Figure 2:**
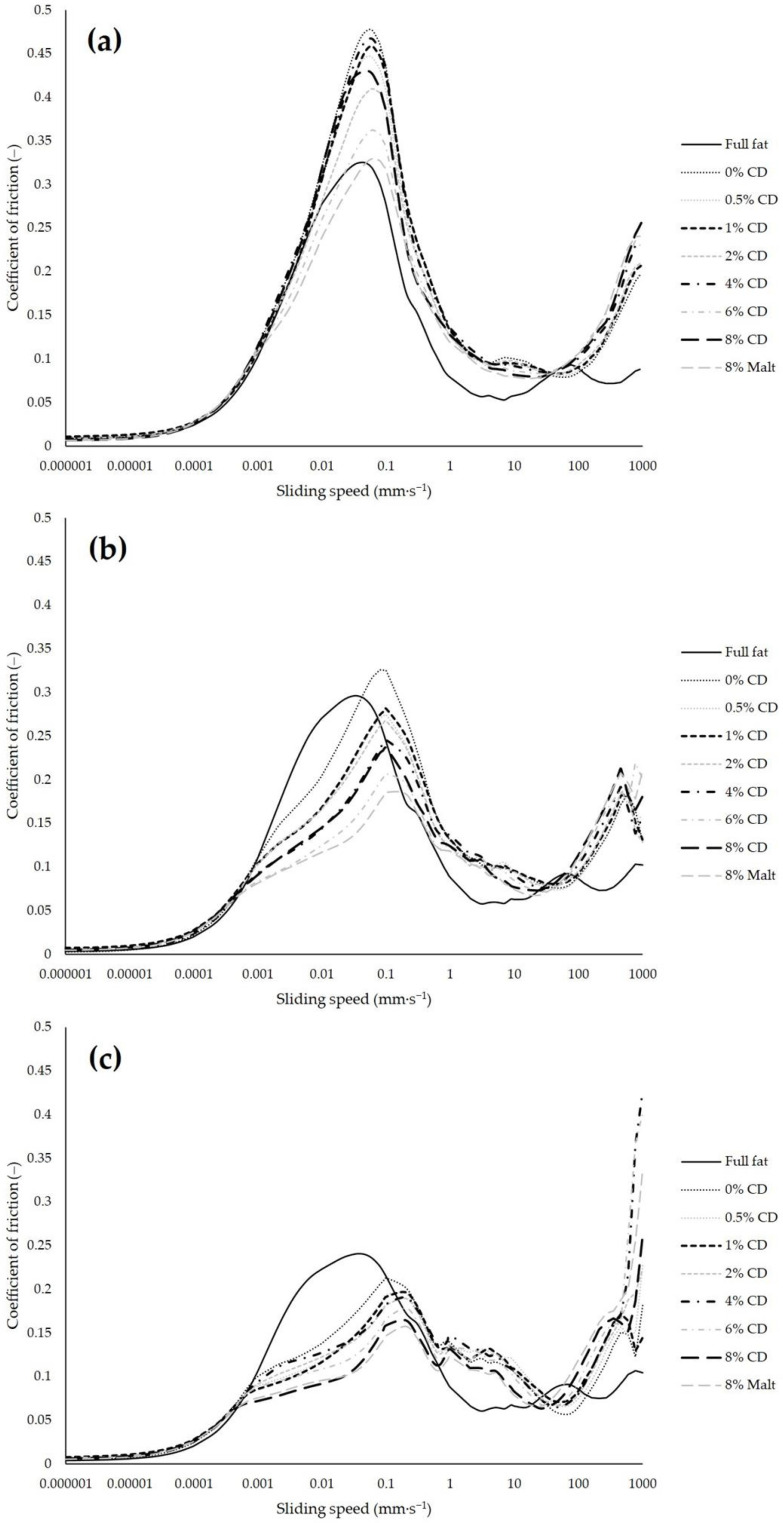
The Stribeck curve 1 (**a**), curve 2 (**b**), and curve 3 (**c**): the coefficient of friction versus the sliding speed of all mayonnaise formulations. The curves are mean values of all four measurements per each batch.

The tribological properties of the mayonnaise samples are described by the coefficient of friction (COF) versus the sliding speed. The lower the COF, the lower the friction between the tribological surfaces, which is due to better lubrication properties of the food sample if the wear of the surfaces is negligible. Figure 2 shows the mean value Stribeck curves of the samples for all tribological measurements. Every measurement generated three Stribeck curves in succession, which are indicated as curve 1, curve 2, and curve 3, respectively. Researchers estimated a speed of up to 200 mm∙s^−1^ for the movement of the tongue during oral processing [16], but to represent the entire Stribeck curve, the speed range was extended to 1000 mm∙s^−1^, even if this is not considered to be representative of oral processing conditions.

There were no noteworthy differences between the COFs of the different formulations at very low speed (<0.0006 mm∙s^−1^). In this static-state region of the Stribeck curve, the friction is mainly influenced by the tribo-pair and less by the lubricant [15]. Therefore, these results were to be expected, since the tribo-pairs were identical for all measurements. In the subsequent boundary friction regime, the friction increased with increasing speeds and, reaching a maximum in the range between 0.01 mm∙s^−1^ and 0.2 mm∙s^−1^ in all curves. Subsequently, a lubricating film began to form, which reduced the friction to a local minimum. In this so-called mixed friction regime, the minimal COF values of the mayonnaise samples were in the range between 4 mm∙s^−1^ and 60 mm∙s^−1^. Up to this point, the COF tended to decrease with increasing CD concentrations, i.e., with higher dry matter content, and these mayonnaise samples thus showed improved lubricating properties. Flamminii et al. [12] also showed lower friction values of hydrocolloid enriched mayonnaise with the higher dry matter content in the formulation and the finer oil droplet distribution in their mayonnaise sample. Interestingly, the samples with 8% CD und 8% Malt, i.e., with the same dry matter content, initially showed different Stribeck curves, and the Malt sample appeared to have better lubricating properties. This indicates that the differences in the COF between the samples with different concentrations cannot be attributed to the different water content alone, but also to the characteristics of the fat replacer. In Figure 2c (curve 3), the Stribeck curves of the two formulations converge; here, the differences between the COF values of the different CD concentrations could possibly be explained by the differences in the water content. The (elasto-) hydrodynamic friction regime, at higher sliding speeds than the mixed friction regime, is mostly governed by the rheological properties of the lubricant [66]. This very phenomenon can be observed in the hydrodynamic regimes of our curves. The higher the viscosity of the sample, the higher the COF was at high sliding speed levels. The viscosity (Table S2), as well as the in-mouth viscosity Kokini OSS (Figure 1), of the samples increased progressively from the full-fat sample to the reduced-fat samples with increasing CD concentrations, and was the highest with 8 g/100 g Malt. The same order can be observed in the Stribeck curves at higher speed levels, except for very high-speed levels of >500 mm∙s^−1^ in curves 2 and 3, where the COF plummeted (curve 2) or increased rapidly (curve 3).

Overall, some regions in the Stribeck curves exhibited very minor differences between the reduced-fat samples, particularly at low-speed levels and at the minimum friction region in the mixed friction regime. The most important differences occurred at the maximum in the boundary regime and, to a lower extent, at high-speed levels (>100 mm∙s^−1^). The third curves exhibited significant differences at very high-speed levels (>700 mm∙s^−1^), which may be due to a larger destruction of the samples. In addition, the COF decreased from curve 1, over curve 2, to curve 3 for each formulation, as did the relative differences between the samples. The wear of the pins can be excluded in this case, because the same pins were used for all four measurements of one sample batch. Additionally, the COF dropped by differing amounts from curve 1 to curve 3 for each formulation. Thus, the decrease can be attributed to the changes in the lubrication properties. According to Pondicherry et al. [15], the frictional behavior of the system for the second and third curves depends on the degree of run-in in the first curve, but also on the formation of a lubricating film on the surfaces and structural changes within the sample. In curve 1, the full-fat sample showed the best lubrication behavior in the range of 0.05 to 30 mm∙s^−1^, at the maximum, and at higher-speed levels (>110 mm∙s^−1^). This phenomenon almost disappeared in curve 2, because the COF of the reduced-fat samples decreased proportionally more strongly from curve 1 to curve 2 than the COF of the full-fat sample. The decrease in the COF between curve 2 and curve 3 was also proportionally larger for the reduced-fat samples compared to the full-fat sample, but overall, not to the same extent as the decrease between curve 1 and curve 2. It is possible that the higher fat content and larger, less stabilized fat droplets in the full-fat mayonnaise tended to coalesce. Thus, a lubricating film might have been produced from the very beginning of the experiment, already showing lower friction in curve 1 and resulting in fewer changes in curve 2 and 3. Furthermore, higher contents of starch, skim milk powder, and xanthan gum in the reduced-fat samples enhanced the stability of the fat droplet distribution in the corresponding emulsion matrix at the beginning (curve 1), resulting in a less-developed fat film and a higher COF. Whereas, with shearing and heating time, the network and structure in the reduced-fat samples were continuously destroyed, and the fat droplets coalesced, interfacing between the surface of the pin and the mayonnaise sample [17] and resulting in lower COF values in the later curves 2 and 3. Dresselhuis et al. [18] also showed that emulsions, which are more prone to coalescence, are associated with lower friction. Thus, the different lubrication behaviors could be explained by the most important determinant of film formation in oil-in-water emulsions: the extent to which oil droplets wet the surface [67,68]. The fat forms a film on the pin surface following a plate-out mechanism [19], inducing the decrease of friction [69,70], which could be observed in the full-fat mayonnaise in the first curve.

A few mechanical engineering researchers applied the dynamic concentration theory to describe the hydrodynamic effects of technical emulsions in the gap between the tribo-pair surfaces [71,72,73,74]. According to this theory, the fat droplet concentration increases dynamically in the gap and an inversion of the emulsion can occur, depending on the tribological system and the adjustments. An oil-in-water emulsion like mayonnaise might invert into a water-in-oil emulsion, with oil becoming the continuous phase, resulting in an enhanced lubrication. Bellamy et al. [68] used this theory to explain the superior lubricating capacity of their emulsions with 33% fat content compared to emulsions with 22% fat content. They suggested that the fat content of 22% did not reach the critical local concentration for an emulsion inversion, confirming their suggestion with a similar interpretation of Vicente et al. [75]. The latter concluded that the formation of the lubricating film depends on the viscosity ratio between the oil and aqueous phases. If the viscosity of the dispersed oil phase is at least 5.8 times more viscous than the continuous aqueous phase, the oil phase controls the formation of the lubricating film. At lower ratios, the aqueous phase dominates the film formation. In our case, the viscosity of the oil phase in the full-fat mayonnaise was much higher than that of the corresponding aqueous phase, and the oil phase dominated the film formation according to this theory. In contrast, in the reduced-fat mayonnaise samples, thickening ingredients like xanthan gum increased the viscosity of the aqueous phase, and shear and heating was necessary to break down the emulsion system, leading to a delayed fat film formation in the tribological measurements. Additionally, the full-fat sample provided larger surface coverage of the hydrophobic PDMS surface and hence, greater lubrication due to the larger volume of fat in the formulation [76].

To decide which curve or which characteristics of the tribological results better represent the actual oral processing of the food, the correlations in Section 3.6 must be considered.

### 3.5. Selected Sensory Attributes

A sensory panel of twelve people ranked the mayonnaise samples in an ascending order of intensity according to the attributes of firmness, stickiness, and creaminess (Table 4). Since all $\chi_{r}^{2}$ values were higher than the critical value $\chi_{crit}^{2}$ = 15.15, the differences between the samples were considered as significant (*α* = 0.05).

The firmness of the samples increased from the full-fat sample to the reduced-fat samples and also with increasing CD concentration, revealing a plateau at concentrations ≥4 g/100 g, as was observed for instrumentally measured firmness. While there was no statistically significant difference in stickiness between higher and lower CD concentrations, the data still indicated trends of higher stickiness with higher CD concentration. A similar trend could be observed for creaminess. These observations will have to be studied further in additional and enhanced sensory tests, e.g., by increasing the panel size, repeating sensory sessions, or reducing the number of formulations assessed. Correlations between sensory and instrumental data are discussed in the following section.

### 3.6. Correlations

**Figure 3:**
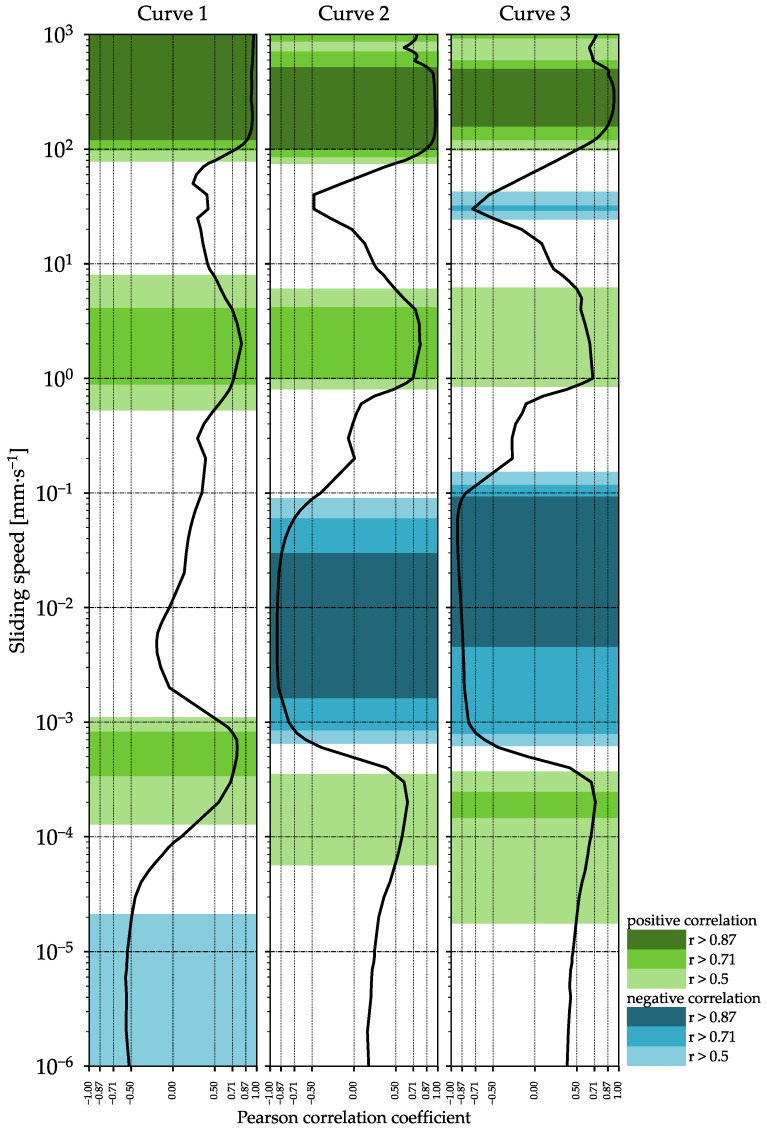
The Pearson correlation coefficient for the sensory attribute of stickiness and the coefficient of friction over the entire sliding speed range for all three curves of the tribological measurements.

In order to determine possible correlations between the results of the various methods, the Pearson correlation coefficient was calculated. The Pearson correlation coefficient between the COF of the samples and the results of the other methods was calculated at individual sliding speeds over the entire speed range. Figure S5 exemplifies the rank sums of the sensory attribute of stickiness over the COF at a sliding speed of 180 mm∙s^−1^ of curve 2 for all nine formulations. The Pearson correlation coefficient for these results was *r* = 0.97, indicating a very high correlation. The higher the COF at the sliding speed of 180 mm∙s^−1^, the higher the sample was ranked in terms of its stickiness properties in the sensory analysis. Therefore, according to the data, a prediction of the sensory analysis would be possible via the evaluation of the COF at a sliding speed of 180 mm∙s^−1^. In order to identify sliding speed ranges where a (very) high correlation between the COF and the sensory attribute of stickiness occurred, the Pearson correlation coefficient was plotted over the sliding speed for all three curves of the tribological measurements (Figure 3). For a better visualization, the speed ranges with high correlations were highlighted in different colors. Figure 3 shows several speed ranges in which the correlation between COF and the sensory attribute of stickiness was high to very high. Furthermore, there were sections with a high correlation in the range provided in the literature for the sliding speed in the mouth (up to 200 mm∙s^−1^). However, other ranges showed even higher correlations and could therefore also serve to predict sensory results, even if they do not precisely match the real conditions in the mouth. The results strongly suggest that there is a correlation between tribological measurements and the sensory analysis of mayonnaise.

In addition to the correlation over the entire speed range, we calculated the Pearson correlation coefficient between the sensory data and the maximum COF in the boundary regime of the tribological measurements. The highest correlation was found between the maximum COF of curve 3 and the sensory attribute of creaminess, with a correlation coefficient of −0.92. The higher the ranking of the mayonnaise samples in the sensory attribute of creaminess, the lower the COF in the maximum, indicating that the maximum COF could be used as a predictor for the perceived creaminess of emulsions. Interestingly, the speed at maximal COF tended to be higher with a higher ranking in creaminess. To mimic the actual oral process in the human mouth more precisely using tribological measurements, saliva plays an important role. Several researchers described an important effect of saliva on the tribological system and the COF. Bellamy et al. [68] obtained larger oil droplets solely by adding saliva to an emulsion, which resulted in the reduction of the COF in tribological measurements. Like other authors, they described an alteration of the lubrication by adding saliva, but there is little literature available indicating whether or not this indeed better represents real oral conditions in tribological measurements and allows higher correlation between instrumental and sensory data [16,77,78,79,80]. This possibility should be investigated in further experiments. Even though the experimental conditions such as the tribological system, the surfaces used, and the measurement parameters are not fully representative of the diversity and complexity of oral conditions, in our study, analogous trends between the sensory results and tribological data were consistent with the results in the literature [68,81].

Table 5 shows the Pearson correlation coefficient between the sensory attributes (stickiness, firmness, creaminess) and the results of rheology (hysteresis area (Figure S4), viscosity, consistency, flow index, Kokini OSS), spreadability (work of shear, work of adhesion) and texture analysis (firmness, stickiness), respectively. Here too, a very high correlation can be observed between the instrumentally collected data and the sensory analysis. The Kokini OSS as the calculated in-mouth thickness showed a very high correlation (*r* = 0.94) with the sensory attribute of creaminess, confirmed by Bayarri et al. [82], who also found a high correlation between the Kokini OSS and the perceived thickness in the mouth. Ross et al. [83] described a very high correlation between the apparent viscosity at a shear rate of 10 s^−1^ and the oral cohesiveness perception, as well as a high correlation between the apparent viscosity at 100 s^−1^ and the oral stickiness perception, respectively, which can be confirmed by our results, although they used hydrocolloid-thickened fluids without emulsified oil in their study. Additionally, as described above, the yield stress determines the perceived thickness or creaminess during mastication [49], which was also found for our mayonnaise samples. The higher the yield stress, the higher the ranking in the sensory analysis for creaminess.

The relationship of instrumental and sensory measurements is not always linear; hence, Stevens [46] suggested the psychophysical power law, later also known as Stevens’ law, to relate a physical stimulus to the perceived sensation [46]. Therefore, the correlations between our results were also evaluated taking Stevens’ power law into account (Table 6).

The exponent *β* indicates the type of the relationship between the sensory and the instrumental data. All values for *β* in Table 6 are greater than one, indicating a non-linear relationship with a more rapid increase of the sensory perception than the instrumental measurement. The correlation coefficient *r* shows that some correlations are better described by a linear and some better by a non-linear relationship of the instrumental and sensory data. Using a power law correlation, the sensory attribute of creaminess can be very well predicted by spreadability, the Kokini OSS, or viscosity. Similarly, the attributes of firmness and stickiness are very well described by the corresponding instrumental firmness and stickiness measurements by applying a power law correlation. An additional cross-correlation between firmness and stickiness can be observed, which was also found by Agyei-Amponsah et al. [17].

Table 7 specifies additional Pearson correlation coefficients between selected results of the experimental series. There is a very high correlation between the consistency in the rheological measurements and the firmness of the texture analysis, which Liu et al. [37] also found for low-fat mayonnaise with different fat mimetics. The correlation between the yield stress and the spreadability (work of shear and work of adhesion) is also very high, confirming the conclusion of Taslikh et al. [49] that the level of yield stress determines the spreadability at the time of utilization.

## 4. Conclusions

The results of the study showed that there are partly very high correlations between instrumental measurement data and the evaluation by the sensory panel. Our results suggest that tribological measurements can replace sensory analysis for the attribute of stickiness. Texture analysis can replace it for the attribute of firmness. In this case, the correlation was better described by a non-linear relationship of the instrumental and sensory data (Stevens’ power law). Furthermore, the correlation between the Kokini OSS and the sensory attribute of creaminess was very high. While it is hard to establish a causal link to explain the correlations, the data showed certain trends. Within the limits of the model, the comparatively easy-to-perform analytical methods have the potential to measure the influence of fat replacers on food emulsions and can predict parts of the sensory analysis. In the future, these evaluation tools could help to estimate the possible applications of new vegetable-fat exchange systems without extensive application and sensory tests. This can contribute to a more systematic and targeted development and significantly shorten the development time for food manufacturers, allowing small and medium-sized enterprises to place innovative and sensory optimized fat- and calorie-reduced products on the market, an opportunity that has so far been reserved almost exclusively for large corporations. Nevertheless, further research is necessary to understand the dynamic and poorly defined oral conditions to be able to better mimic them using instrumental analysis such as tribology. Furthermore, the sensory tests could be significantly enhanced to provide more detailed and comprehensive data to gain a more holistic picture of the sensory properties.

## Figures and Tables

**Table 1:** Indicative values of the glycosidic bond distribution (in %), respectively, in (1) corn dextrin, (2) standard maltodextrin, and (3) starch, according to Lefranc-Millot [10]. Reproduced with permission from C. Lefranc-Millot, NUTRIOSE® 06: a useful soluble dietary fibre for added nutritional value; published by John Wiley and Sons, 2008.

Type of Glycosidic Linkages	(1)	(2)	(3)
(1,4)	41	95	95
(1,6)	32	5	5
(1,2)	13	0	0
(1,3)	14	0	0

**Table 2:** Formulations of the mayonnaise samples (CD = corn dextrin, Malt = maltodextrin).

	Full Fat	0% CD	0.5% CD	1% CD	2% CD	4% CD	6% CD	8% CD	8% Malt
	(g/100 g)	(g/100 g)	(g/100 g)	(g/100 g)	(g/100 g)	(g/100 g)	(g/100 g)	(g/100 g)	(g/100 g)
Rapeseed oil	50.3	25	25	25	25	25	25	25	25
Water	37.68	56.38	55.88	55.38	54.38	52.38	50.38	48.38	48.38
Sunflower protein	2	1.2	1.2	1.2	1.2	1.2	1.2	1.2	1.2
Corn starch	1	3	3	3	3	3	3	3	3
Corn dextrin	0	0	0.5	1	2	4	6	8	0
Maltodextrin	0	0	0	0	0	0	0	0	8
Skim milk powder	0	5	5	5	5	5	5	5	5
Xanthan gum	0	0.3	0.3	0.3	0.3	0.3	0.3	0.3	0.3
Citric acid	0.12	0.22	0.22	0.22	0.22	0.22	0.22	0.22	0.22
Sugar	3.7	3.7	3.7	3.7	3.7	3.7	3.7	3.7	3.7
Salt	0.6	0.6	0.6	0.6	0.6	0.6	0.6	0.6	0.6
Mustard	1.6	1.6	1.6	1.6	1.6	1.6	1.6	1.6	1.6
Vinegar	3	3	3	3	3	3	3	3	3

**Table 3:** Firmness, stickiness, work of shear, and work of adhesion (CD = corn dextrin, Malt = maltodextrin).

Formulation	Firmness (mN)	Stickiness (mN)	Work of Shear (N∙s)	Work of Adhesion (N∙s)
Full fat	93.7 ± 2.8 ^d^	−74.1 ± 3.1 ^a^	1.28 ± 0.09 ^e^	−0.40 ± 0.03 ^a^
0% CD	136.0 ± 3.1 ^c^	−126.3 ± 3.0 ^b^	2.38 ± 0.07 ^d^	−0.86 ± 0.04 ^b^
0.5% CD	138.3 ± 4.7 ^c^	−128.3 ± 3.9 ^b^	2.44 ± 0.13 ^cd^	−0.88 ± 0.05 ^b^
1% CD	141.6 ± 4.9 ^bc^	−131.1 ± 5.7 ^bc^	2.44 ± 0.07 ^cd^	−0.88 ± 0.03 ^b^
2% CD	144.6 ± 7.0 ^b^	−135.9 ± 6.8 ^c^	2.54 ± 0.20 ^c^	−0.92 ± 0.08 ^b^
4% CD	159.6 ± 5.1 ^a^	−149.8 ± 5.9 ^d^	2.89 ± 0.16 ^b^	−1.05 ± 0.06 ^c^
6% CD	157.9 ± 10.4 ^a^	−149.1 ± 10.8 ^d^	2.92 ± 0.23 ^b^	−1.04 ± 0.10 ^c^
8% CD	156.0 ± 12.3 ^a^	−150.2 ± 13.1 ^d^	3.00 ± 0.26 ^ab^	−1.09 ± 0.12 ^cd^
8% Malt	157.8 ± 9.3 ^a^	−154.3 ± 9.6 ^d^	3.12 ± 0.17 ^a^	−1.15 ± 0.05 ^d^

The data are expressed as mean ± standard deviation (*n* > 22). Values followed by different letters in a column indicate significant differences between samples (*p* < 0.05) following one-way ANOVA (Tukey).

**Table 4:** Rank sum scores for the sensory attributes of firmness, stickiness, and creaminess and the critical $\chi_{r}^{2}$ value.

Formulation	Firmness	Stickiness	Creaminess
Full fat	20 ^e^	16 ^d^	19 ^d^
0% CD	38 ^de^	45 ^bc^	52 ^bc^
0.5% CD	43 ^de^	42 ^c^	49 ^c^
1% CD	46 ^cd^	57 ^abc^	49 ^c^
2% CD	52 ^bcd^	55 ^abc^	52 ^bc^
4% CD	70 ^abc^	71 ^a^	62 ^abc^
6% CD	82 ^a^	64 ^abc^	58 ^abc^
8% CD	73 ^ab^	75 ^a^	76 ^ab^
8% Malt	71 ^abc^	70 ^ab^	78 ^a^
$\chi_{r}^{2}$	39.8	33.4	29.3

Values followed by different letters in a column indicate significant differences between samples following two-way Friedman analysis and the least significant difference (LSD) test (*α* = 0.05).

**Table 5:** The Pearson correlation coefficient between the sensory attributes of stickiness, firmness, and creaminess, and the results of rheology, texture analysis, and spreadability measurements.

	Sensory Stickiness	Sensory Firmness	Sensory Creaminess
Hysteresis area	0.92	0.94	0.94
Viscosity at 10 s^−1^	0.94	0.89	0.95
Viscosity at 100 s^−1^	0.93	0.87	0.94
Yield stress	0.89	0.84	0.91
Consistency	0.94	0.87	0.91
Kokini OSS	0.94	0.89	0.94
Work of shear	0.96	0.92	0.96
Work of adhesion	−0.95	−0.89	−0.96
Firmness	0.96	0.91	0.91
Stickiness	−0.96	−0.90	−0.93

**Table 6:** The relationship between sensory and instrumental data using Stevens’ law, with the constant *k*, the exponent *β*, and the correlation coefficient *r*.

	Sensory Stickiness			Sensory Firmness			Sensory Creaminess		
	*k*	*β*	*r*	*k*	*β*	*r*	*k*	*β*	*r*
Hysteresis area	0.041	1.30	0.91	0.015	1.48	0.93	0.045	1.28	0.93
Viscosity at 10 s^−1^	1.20 × 10^−5^	1.60	0.95	9.91 × 10^−7^	1.85	0.92	2.35 × 10^−5^	1.53	0.95
Viscosity at 100 s^−1^	1.53×10^−3^	1.33	0.93	1.55 × 10^−4^	1.62	0.89	1.76 × 10^−3^	1.31	0.95
Yield stress	0.28	1.26	0.88	0.19	1.36	0.83	0.27	1.27	0.91
Consistency	0.19	1.61	0.95	0.017	2.38	0.94	0.57	1.30	0.91
Kokini OSS	0.022	1.55	0.94	5.38 × 10^−3^	1.83	0.91	0.029	1.50	0.95
Work of shear	10.91	1.70	0.96	7.69	2.04	0.94	11.87	1.61	0.97
Work of adhesion	61.26	1.52	0.96	61.34	1.80	0.92	61.01	1.48	0.97
Firmness	3.81 × 10^−5^	2.85	0.97	9.97 × 10^−7^	3.58	0.96	4.37 × 10^−4^	2.36	0.91
Stickiness	9.21 × 10^−4^	2.24	0.97	5.27 × 10^−5^	2.82	0.95	3.4 × 10^−3^	1.97	0.94

**Table 7:** The Pearson correlation coefficient between various results of the experimental series.

	Pearson Correlation Coefficient *r*
Yield stress and work of shear	0.94
Yield stress and work of adhesion	−0.94
Consistency and firmness	0.99
Viscosity at 10 s^−1^ and work of shear	1.00
Viscosity at 10 s^−1^ and firmness	0.99
Viscosity at 10 s^−1^ and sliding speed at maximum coefficient of friction (of curve 2/curve 3)	0.96/0.93
Viscosity at 10 s^−1^ and maximum COF of curve 3	−0.89

## Data Availability

The data presented in this study are available on request from the corresponding author.

## Supplementary Materials

The following supporting information can be downloaded at: https://www.mdpi.com/article/10.3390/foods11060806/s1, Table S1: The composition of the samples based on 100 g mayonnaise (abs = absolute, DM = dry matter, CD = corn dextrin, Malt = maltodextrin), Table S2: The viscosity at a shear rate of 10 s^−1^ and 100 s^−1^, respectively, as well as the yield stress, consistency, and flow index (Herschel-Bulkley model) of the rheological characterization of the mayonnaise samples, Figure S1: The force-time diagram of four single measurements as example curves for the texture analysis (CD = corn dextrin), Figure S2: The force-time diagram of four single measurements as example curves for the spreadability analysis, Figure S3: The flow curve diagram of four single measurements as example curves for the rheological analysis (arrows indicates curve progression), Figure S4: The hysteresis area of the mayonnaise samples determined by rheological measurements. The data are expressed as mean ± standard deviation (*n* > 9). Bars with different letters indicate significant differences between samples (*p* < 0.05) following one-way ANOVA (Tukey), Figure S5: The rank sum scores for the sensory attribute of stickiness over the coefficient of friction at a sliding speed of 180 mm·s^−1^ (curve 2) for all nine formulations (error bars represent the standard deviation of the tribological measurements).
