# Supplementary material for: Characterization of Reduced-Fat Mayonnaise and Comparison of Sensory Perception, Rheological, Tribological, and Textural Analyses
Source: Foods. 2022 Mar 11;11(6):806. doi: 10.3390/foods11060806 (PMC8954533; doi:10.3390/foods11060806)
Supplement: Supplementary file 1 [file foods-11-00806-s001.zip › foods-1596412-supplementary.pdf]

Supplementary Materials

Table S1. The composition of the samples based on 100 g mayonnaise (abs = absolute, DM = dry matter, CD = corn dextrin, Malt = maltodextrin).

Formulation	DM	Fat (abs)	Fat (in DM)	Protein (abs)	Protein (in DM)	Energy Density [1]	Energy Density [1]
**per 100 g**	**(g)**	**(g)**	**(g)**	**(g)**	**(g)**	**(kJ)**	**(kcal)**
Full fat	59.5 ± 0.8 ^{a}	50.9 ± 1.7 ^{a}	85.6 ± 1.8 ^{a}	1.1 ± 0.1 ^{b}	1.9 ± 0.1 ^{f}	1816	441
0% CD	41.3 ± 0.2 ^{b}	26.5 ± 0.3 ^{b}	64.2 ± 0.8 ^{b}	2.7 ± 0.0 ^{a}	6.5 ± 0.1 ^{e}	1057	256
0.5% CD	42.1 ± 0.6 ^{bc}	27.2 ± 1.1 ^{b}	64.6 ± 3.1 ^{b}	2.6 ± 0.0 ^{a}	6.2 ± 0.1 ^{de}	1061	257
1% CD	42.6 ± 0.5 ^{bc}	26.4 ± 0.3 ^{b}	62.0 ± 0.5 ^{bc}	2.6 ± 0.0 ^{a}	6.1 ± 0.0 ^{cd}	1065	258
2% CD	43.6 ± 0.3 ^{c}	26.7 ± 0.5 ^{b}	61.3 ± 1.6 ^{bc}	2.6 ± 0.1 ^{a}	5.9 ± 0.2 ^{bc}	1074	260
4% CD	45.5 ± 0.6 ^{d}	26.4 ± 0.4 ^{b}	57.9 ± 0.2 ^{cd}	2.6 ± 0.1 ^{a}	5.6 ± 0.1 ^{b}	1091	264
6% CD	49.0 ± 1.3 ^{e}	26.7 ± 0.7 ^{b}	54.4 ± 1.2 ^{de}	2.6 ± 0.0 ^{a}	5.3 ± 0.1 ^{a}	1107	268
8% CD	50.7 ± 0.3 ^{e}	26.0 ± 0.5 ^{b}	51.2 ± 0.9 ^{e}	2.6 ± 0.0 ^{a}	5.1 ± 0.1 ^{a}	1124	272
8% Malt	49.7 ± 0.1 ^{e}	28.0 ± 0.5 ^{b}	56.5 ± 1.0 ^{d}	2.6 ± 0.0 ^{a}	5.3 ± 0.1 ^{a}	1184	286

[1] Energy density was calculated using the manufacturer specifications of the ingredients and the respective formulations of the samples. The data are expressed as mean ± standard deviation (n = 9). Values followed by different letters in a column indicate significant differences between samples ($p < 0.05$) following one-way ANOVA (Tukey).

Table S2. The viscosity at a shear rate of 10 s^{-1} and 100 s^{-1}, respectively, as well as the yield stress, consistency, and flow index (Herschel–Bulkley model) of the rheological characterization of the mayonnaise samples.

Formulation	Viscosity η at 10 s^{-1} (Pa·s)	Viscosity η at 100 s^{-1} (Pa·s)	Yield Stress τ_0 (Pa)	Consistency K (Pa·s^n)	Flow index n (–)
Full fat	6.8 ± 0.2 ^{e}	1.0 ± 0.1 ^{e}	35.1 ± 5.1 ^{e}	12.6 ± 3.8 ^{c}	0.37 ± 0.04 ^{b}
0% CD	13.7 ± 0.3 ^{d}	2.5 ± 0.0 ^{d}	59.0 ± 6.1 ^{d}	31.2 ± 4.8 ^{b}	0.39 ± 0.03 ^{ab}
0.5% CD	14.1 ± 0.4 ^{cd}	2.6 ± 0.1 ^{cd}	57.8 ± 5.1 ^{d}	33.7 ± 4.4 ^{ab}	0.39 ± 0.02 ^{b}
1% CD	14.2 ± 0.4 ^{cd}	2.6 ± 0.0 ^{cd}	63.9 ± 5.8 ^{cd}	32.0 ± 5.3 ^{ab}	0.39 ± 0.03 ^{ab}
2% CD	15.0 ± 0.7 ^{c}	2.7 ± 0.1 ^{c}	70.2 ± 4.2 ^{bc}	34.7 ± 3.8 ^{ab}	0.39 ± 0.02 ^{ab}
4% CD	16.6 ± 0.5 ^{b}	3.0 ± 0.1 ^{b}	69.5 ± 4.5 ^{bc}	39.8 ± 4.6 ^{a}	0.38 ± 0.02 ^{b}
6% CD	16.7 ± 1.2 ^{b}	3.0 ± 0.2 ^{b}	72.8 ± 5.2 ^{b}	38.6 ± 7.3 ^{a}	0.40 ± 0.03 ^{ab}
8% CD	16.7 ± 1.1 ^{b}	3.0 ± 0.2 ^{b}	70.1 ± 8.2 ^{bc}	39.6 ± 3.4 ^{a}	0.39 ± 0.01 ^{b}
8% Malt	18.3 ± 0.9 ^{a}	3.4 ± 0.1 ^{a}	88.8 ± 5.5 ^{a}	38.4 ± 5.5 ^{ab}	0.44 ± 0.01 ^{a}

The data are expressed as mean ± standard deviation (n = 9). Values followed by different letters in a column indicate significant differences between samples ($p < 0.05$) following one-way ANOVA (Tukey).

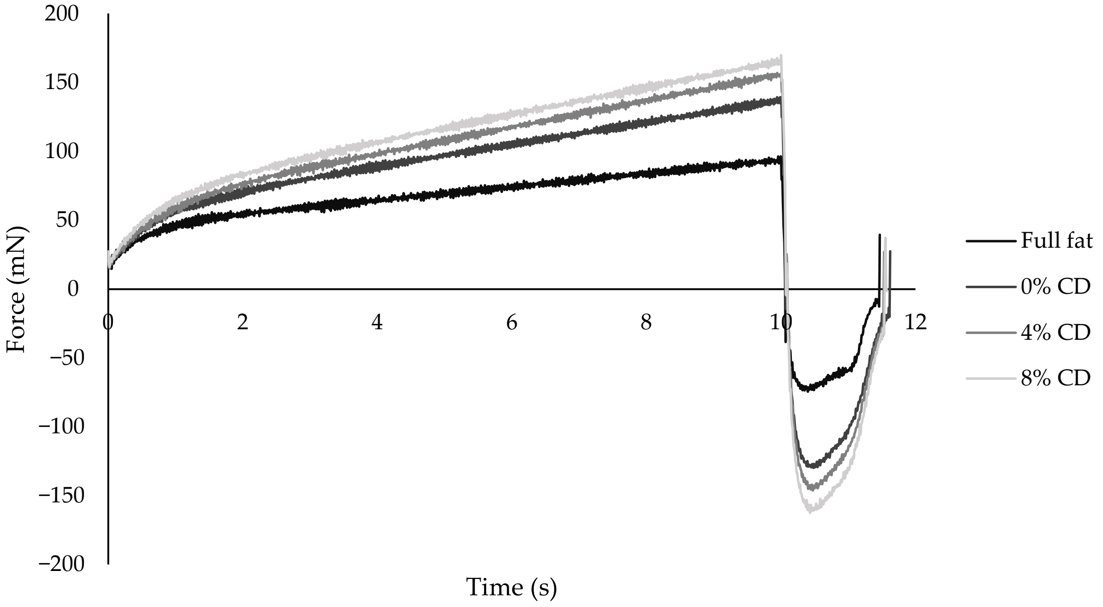

Figure S1. The force-time diagram of four single measurements as example curves for the texture analysis (CD = corn dextrin).

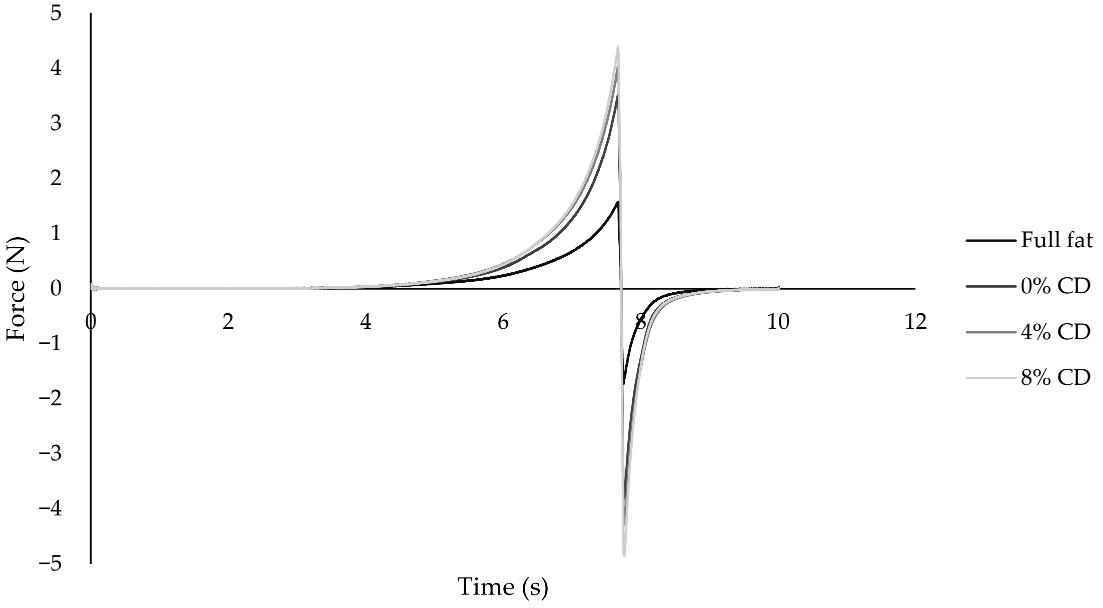

Figure S2. The force-time diagram of four single measurements as example curves for the spreadability analysis.

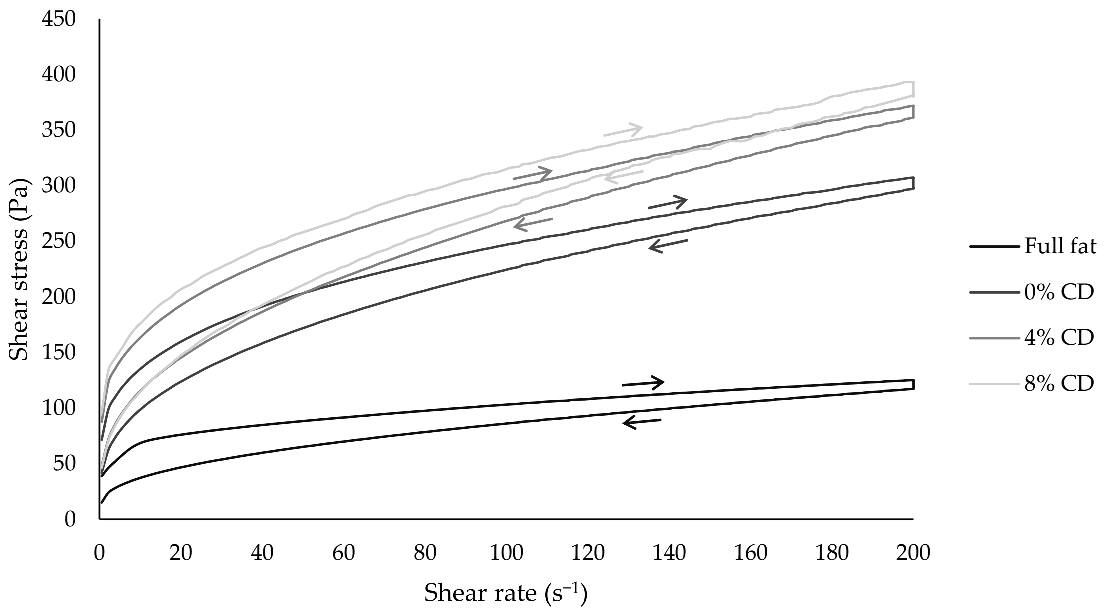

Figure S3. The flow curve diagram of four single measurements as example curves for the rheological analysis (arrows indicates curve progression).

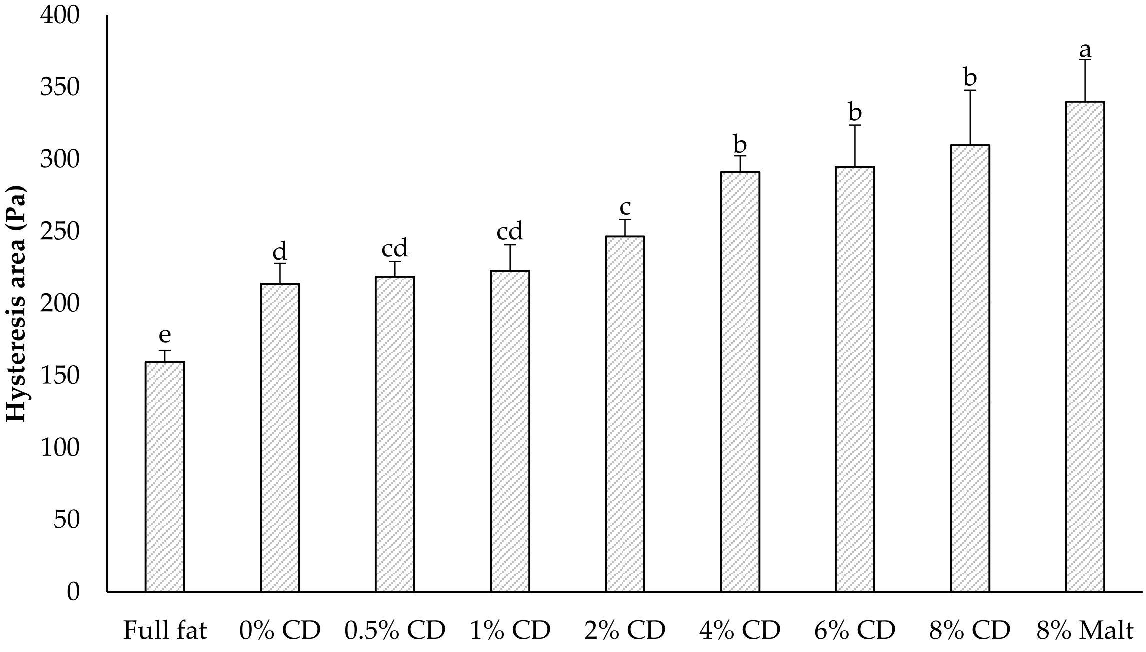

Figure S4. The hysteresis area of the mayonnaise samples determined by rheological measurements. The data are expressed as mean ± standard deviation ($n > 9$). Bars with different letters indicate significant differences between samples ($p < 0.05$) following one-way ANOVA (Tukey).

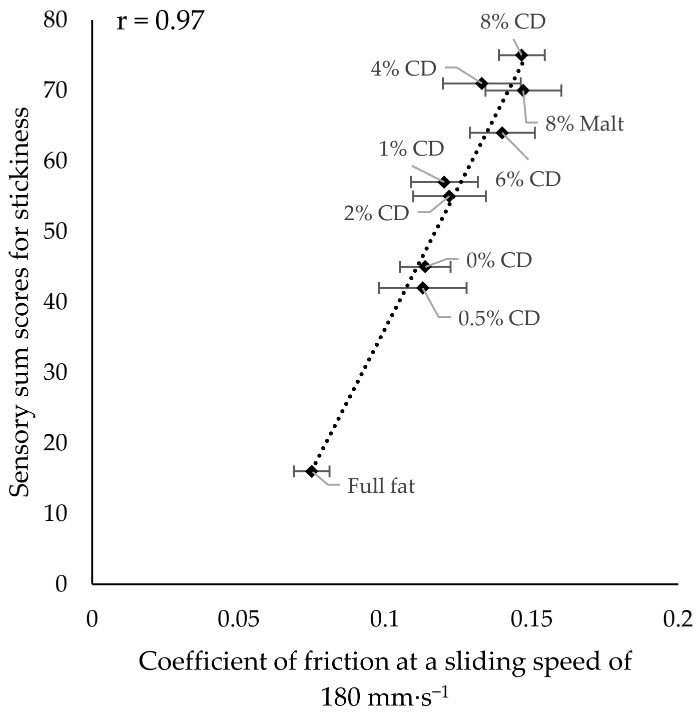

Figure S5. The rank sum scores for the sensory attribute of stickiness over the coefficient of friction at a sliding speed of 180 mm·s^{-1} (curve 2) for all nine formulations (error bars represent the standard deviation of the tribological measurements).
